# Supplementary material for: Production and Characterization of Peptide Antibodies to the C-Terminal of Frameshifted Calreticulin Associated with Myeloproliferative Diseases
Source: Int J Mol Sci. 2022 Jun 18;23(12):6803. doi: 10.3390/ijms23126803 (PMC9223637; doi:10.3390/ijms23126803)
Supplement: Supplementary file 1 [file ijms-23-06803-s001.zip › ijms-1695551-supplementary.pptx]

## Slide 1
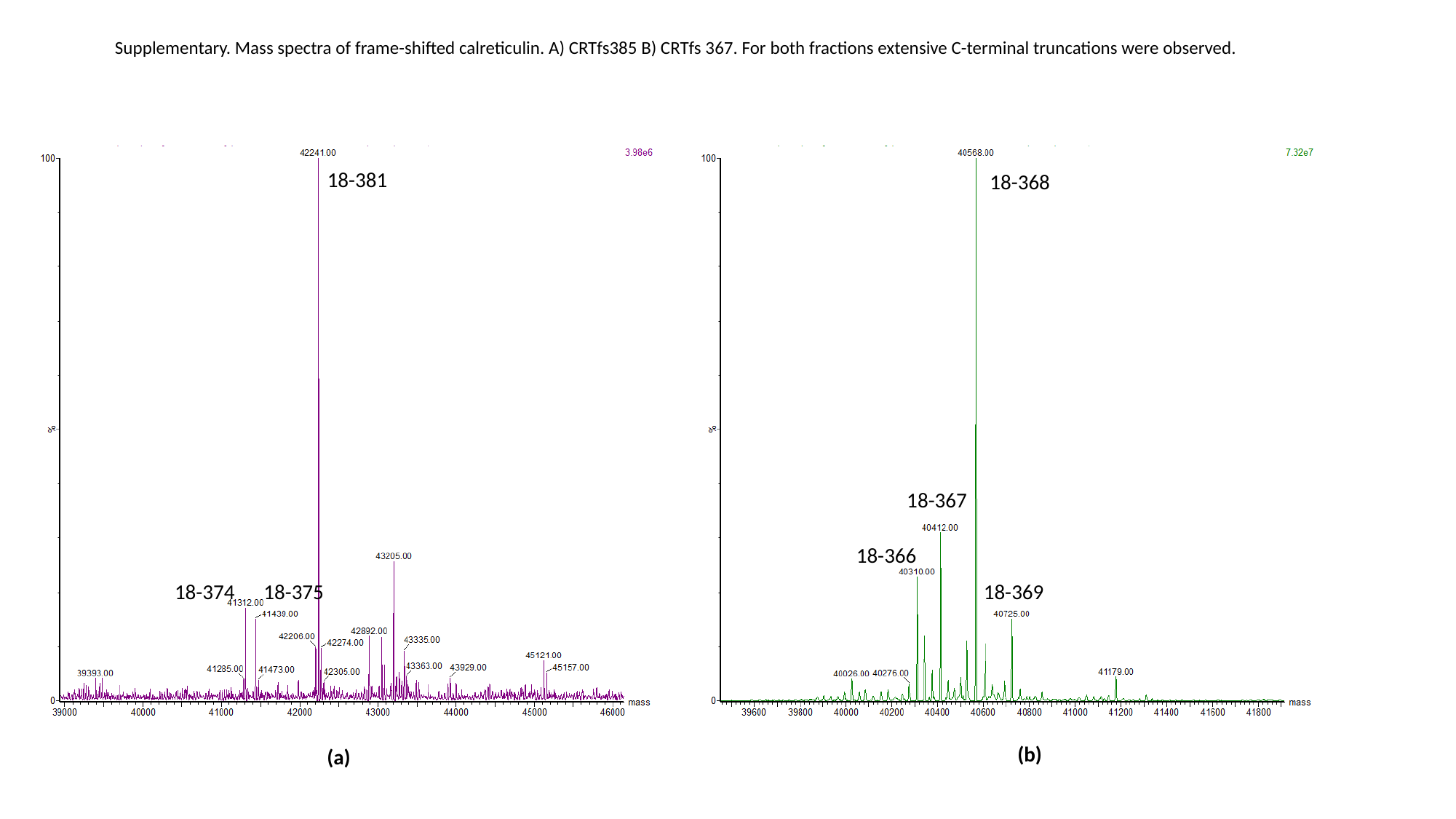

Supplementary. Mass spectra of frame-shifted calreticulin. A) CRTfs385 B) CRTfs 367. For both fractions extensive C-terminal truncations were observed.
18-381
18-368
18-367
18-366
18-369
18-374
18-375
(b)
(a)
